# Automatic Segmentation of Lumbar Spine MRI Images Based on Improved Attention U-Net

**DOI:** 10.1155/2022/4259471

**Published:** 2022-09-14

**Authors:** Shuai Wang, Zhengwei Jiang, Hualin Yang, Xiangrong Li, Zhicheng Yang

**Affiliations:** ^1^College of Mechanical and Electrical Engineering, Qingdao University of Science and Technology, Qingdao 266061, China; ^2^Department of Radiology, Qilu Hospital (Qingdao), Cheeloo College of Medicine, Shandong University, Qingdao, China

## Abstract

Lumbar spine segmentation is important to help doctors diagnose lumbar disc herniation (LDH) and patients' rehabilitation treatment. In order to accurately segment the lumbar spine, a lumbar spine image segmentation algorithm based on improved Attention U-Net is proposed. The algorithm is based on Attention U-Net, the attention module based on multilevel feature map fusion is adopted, two residual modules are introduced instead of the original convolution blocks. a hybrid loss function is used for prediction during the training process, and finally, the image superposition process is realized. In this experiment, we expanded 420 lumbar MRI images of 180 patients to 1000 images and trained them by different algorithms, respectively, and accuracy, recall, and Dice similarity coefficient metrics were used to analyze these algorithms. The results show that compared with SVM, FCN, R-CNN, U-Net, and Attention U-Net models, the improved model achieved better results in all three evaluations, with 95.50%, 94.53%, and 95.01%, respectively, which proves the better performance of the proposed method for segmentation in lumbar disc and caudal vertebrae.

## 1. Introduction

In recent years, lumbar spine-related diseases have been affecting people's normal work and lives, and some families are bearing a huge economic burden. Lumbar spine diseases mainly include disc herniation (LDH) and lumbar spinal stenosis [1]. As LDH is mainly caused by disc degeneration or overwork, it has the highest prevalence in the age group of 30–50 years old, with a prevalence ratio of 2 : 1.1 between men and women, and 90% of the elderly over 60 years old worldwide suffer from degenerative disc symptoms [2–5]. Due to the increased pressure in life, more and more young people are also suffering from lumbar spine diseases. Lumbar spine diseases are diagnosed by physicians by examining the relevant parts of the lumbar spine. The imaging modalities mainly include computed tomography (CT) [6], magnetic resonance imaging (MRI) [7], and so on. The images with better imaging are selected from lots of CT or MRI images by physicians, and the possible lesions are diagnosed by physicians. As the number of patients increases, the cumbersome diagnostic approach not only increases the consultation time of patients but also puts tremendous work pressure on physicians. Therefore, lumbar segmentation plays an important part in the whole diagnostic process, which helps doctors to observe medical images quickly and accurately, and it facilitates the patient's further treatment.

Medical image segmentation techniques can be divided into two categories: traditional segmentation techniques and deep learning-based methods. The former includes threshold-based segmentation [8], edge-based segmentation [9], region-based segmentation [10, 11], and active contour model-based techniques [12, 13], and the latter is mainly neural network-based segmentation [14–18]. Earlier studies have been applied to the lumbar spine segmentation by traditional segmentation techniques. Hoad et al. [19] used a traditional threshold segmentation method applied to the spine MRI images to segment lumbar discs from soft tissues, thereby realizing the computer-aided diagnosis of the spine. Armato et al. [20] demonstrated a random forest based method to extract vertebral height and width, which solved the problem of biomarkers of the lumbar spine. Punarselvam et al. [21] used a watershed method to detect boundaries and edges in the lumbar spine images. Later, this method was added with statistical and spectral texture features by some scholars [22], and they used this method to effectively distinguish the closed area of the intervertebral disc in the image. In recent years, deep learning has shown great advantages in medical image processing. Lee et al. [23] established a reference framework for segmenting lumbar arch roots in CT images, which obtained segmented vertebrae and canal references by using 2D dynamic thresholding and a combined cost based on sparing and finally achieved edge segmentation of the spine. Deng et al. [24] proposed a method based on the combination of contour transform and artificial neural network (ANN). This approach used contour transform to decompose images to obtain contour coefficients and used the ANN to optimize the coefficients of contour transform, thereby improving the performance of lumbar image segmentation. In addition, the method of the deep network with full convolution (FCN) was proposed in some studies [25, 26], which segmented and labeled the lumbar spine at once by using the local lumbar spine environment. FCN combined with the convolutional neural network (CNN) again to improve the segmentation effect, compared the segmentation results with conventional segmentation methods, and the results showed that the segmentation accuracy and efficiency were improved [27]. However, deep convolutional networks are not the only option for medical image segmentation, which requires training with a large amount of data. With the expansion of machine learning, image segmentation has developed different types of models based on full convolutional networks, such as U-Net [28–30], PSPNet [31, 32], and DeepLab [33, 34]. Sunetra et al. [35] showed the LDS U-Net structure to segment ultrasound spine lateral bony features from noisy images, which required only a small number of medical images for training. Saenz-Gamboa et al. [36] used a variant of U-Net for automatic segmentation of lumbar spine MRI images; this model classified labels to each pixel of the image. For the image noise problem of lumbar spine segmentation, Yang et al. [37] showed an automatic initialization level set method based on regional correlation, which introduced the histogram information inside and outside the level set contour, and Tang et al. [38] used a double densely connected U-neural network. This method improved the contrast of vertebral body edges, spinal ducts, and cloudy sacs while reducing image noise.

**Figure 1:**
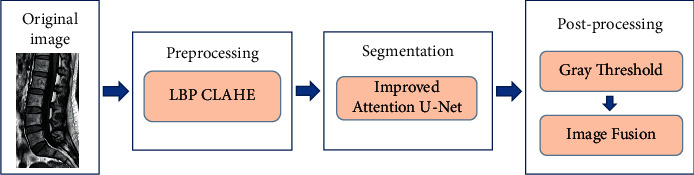
Segmentation process of lumbar spine image.

The lumbar spine can be extracted from the soft tissue by the above lumbar spine segmentation methods, but the accuracy is still slightly low, and the segmentation effect is influenced by the lesion area, as well as the parameter settings, which has some limitations. To solve these problems, this paper proposes a lumbar spine segmentation method with an improved Attention U-Net, which improves the structure of the attention module and residual network. A hybrid loss function is used to improve the detection accuracy. The specific experimental procedure is shown in Figure 1. First, the images are preprocessed by extracting local binary pattern (LBP) features and using contrast limited adaptive histogram equalization (CLAHE). Then, segmentation is performed using a modified Attention U-Net model. Finally, the vertebral blocks and intervertebral discs are extracted by gray threshold, and the images are superimposed by image fusion. MRI is the mainstay of lumbar spine image diagnosis at present. Compared with CT, MRI images have clearer soft tissue contours and have a better effect for imaging intervertebral disc degeneration, so we select MRI images to carry out the experiment of lumbar spine segmentation.

The rest of this article is organized as follows: in Section 2, U-Net and Attention U-Net are introduced, and how to improve Attention U-Net is described in detail. The arrangement of the experiments and the evaluation metrics are described in Section 3. The results of the experiments and postprocessing of the images are described in Section 4. Finally, Section 5 draws conclusions and proposes future research.

## 2. Methods

### 2.1. U-Net and Attention U-Net

U-Net is a convolutional neural network architecture with a simple structure and high efficiency. The architecture consists of two parts: an encoder and a decoder. The encoder part uses convolution and pooling to downsample the image, which doubles the number of feature channels and halves the image size. This part consists of two convolutional layers with 3×3 filters and a 2×2 maximum pooling layer with a step size of 2. ReLU is used for the activation function. The decoder part upsamples the feature image by deconvolution, which reduces the number of features channels and increases the image size, and finally outputs an image of the same size as the original image. It mainly consists of a deconvolutional layer with 2×2 filter and two 3×3 convolutional layers and still uses the ReLU activation function. The feature stitching of U-Net has a better processing effect for problems such as the difficulty in distinguishing biological tissue structures and the display of low-level and high-level features [39]. In addition, the experimental data of some medical images are generally so less that they are not suitable for the complex and large networks, while the U-Net with its simple structure can be better processed for these medical images.

Attention U-Net is a network structure based on U-Net with an added attention mechanism [40]. Compared with U-Net, an attention mechanism is added to the feature map in the encoder part before splicing in the decoder part, so that irrelevant background regions are suppressed and target regions are enhanced. In the lumbar segmentation, the vertebral body, intervertebral disk, and sacral regions are enhanced by the attention mechanism, while the soft tissue regions are suppressed [41]. The oversegmentation of images by the network structure can be effectively reduced by the attention mechanism.

### 2.2. Improved Attention U-Net

**Figure 2:**
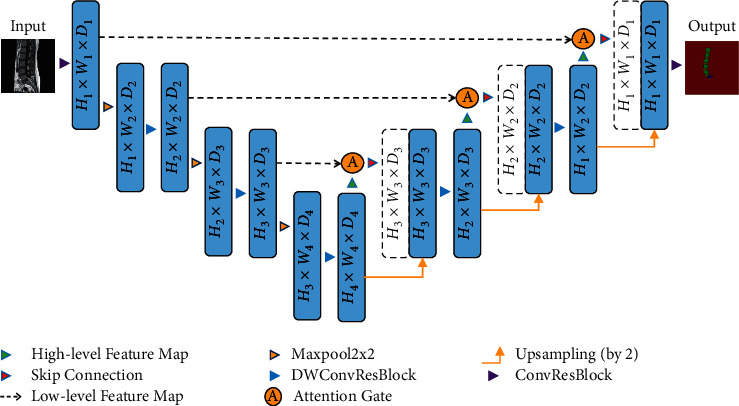
The network structure of improved Attention U-Net.

In this study, an improved network structure is proposed based on Attention U-Net, as shown in Figure 2. The network is presented as U-shaped, with the encoder part on the left and the decoder part on the right side. The number of channels, height, and width of the tensor are denoted by D, H, and W, respectively. Compared with the traditional Attention U-Net, the network framework is built by deep convolution in the bottom feature layer and the top feature layer. Improved residual structure is added to the convolution process of each layer to increase the depth and feature fusion ability of the network. Layers 1–3 in the encoding process and layers 6–8 in the decoding process are connected by jump connections, so that the encoder part is used to generate feature information at different scales in the whole network. An improved attention module is introduced for each jump connection that allows the model to acquire local information more accurately. Finally, the structure reduces the number of four downsampling layers in the traditional U-Net to three, which reduces the number of parameters in the network; therefore, the computational complexity is reduced, which facilitates the acquisition of global features.

#### 2.2.1. Improved Multilevel Attention Module

The attention mechanism is generally applied in the dynamic analysis of vision and classification of images and later in segmentation of images. In image segmentation, the attention mechanism is used to remove redundant information from layers to improve the running speed and segmentation performance of matrix algorithms [42, 43]. The expression of this attention mechanism is

(1) $$\begin{array}{c} AG\left(x,g,g\right)=\sigma \left({Conv}_{2\;d}^{1\times 1}\left(ReLu\left({Conv}_{2\;d}^{1\times 1}\left(Up\left(g\right)\right)\times {Conv}_{2\;d}^{1\times 1}\left(x\right)\right)\right)\right)\times x, \end{array}$$

where the eigengraphs of the encoder output and the gating signal are represented as *x* and g, respectively, *σ* is the sigmoid function, Up represents the upsampling, and Conv_2 d_^1×1^represents the two-dimensional 1 × 1 convolution.

**Figure 3:**
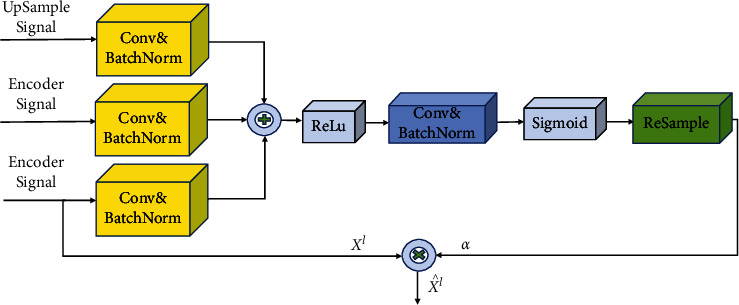
Improved attention module.

According to the characteristics of attention, an attention module based on a multilevel feature map fusion is improved in this study. The improved attention module is shown in Figure 3; in the input phase, the matrix of the encoder part is normalized by conv1×1 and batch normalization (BN) operation in the input stage, combined with the matrix in the upsampling that has undergone convolution and batch normalization, and then, the convergence of the attention parameters is trained by processing the ReLU activation function, the conv1 × 1, and sigmoid activation functions. The attention coefficients *α* (i.e., attention weights) are obtained by resampling. Finally, the output *α* is multiplied with the feature layer of the encoder to obtain the result.

#### 2.2.2. Improved Residual Module

Different from the conventional convolutional neural network, the ResNet [44] residual network deepens the number of network layers through shortcut connections. It still has better running speed and results without adding parameters and data calculations, which can effectively solve the gradient dissipation problem caused by too many output features. The residual network is composed of residual modules, which are as follows:

(2) $$\begin{array}{c} y=F\left(x,\left\{w_{i}\right\}\right)+w_{j}x\text{,} \end{array}$$

where *y* and *x* are the output and input vectors of the residual module, *F*(*x*, {*w*_*i*_}) is the residual mapping, and linear projection *w*_*j*_ is used for matching dimensions in shortcut connection.

This study designs two different residual modules based on the ResNet network structure to replace the convolutional blocks in Attention U-Net, and the module structures are shown in Figures 3 and4.

**Figure 4:**
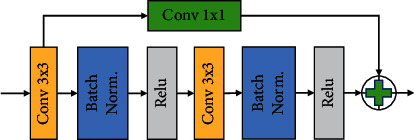
Improved standard convolutional residual blocks.

The convolutional module in Figure 4 is used for feature extraction in the first and last layers of Attention U-Net, adding the underlying residual structure to better extract information in large size and shallow-depth feature maps. Its two convolutional layers use a 3 × 3 convolutional kernel and ReLU function, and the input feature map after 1 × 1 convolution is subjected to a feature summation operation with the output after 3 × 3 convolution. In addition, a batch normalization (BN) operation is performed before using ReLU to speed up the convergence of the model.

**Figure 5:**
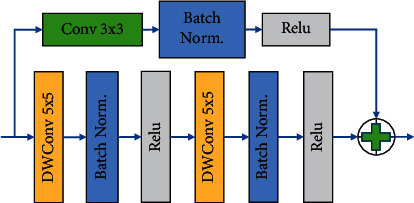
Improved deep convolutional residual blocks.

An improved deep convolutional residual module is shown in Figure 5. It mainly contains two 5 × 5 convolutional layers, a 3 × 3 convolution, and some basic operations. For the feature map that is input to the residual module, the feature map after two 5 × 5 deep convolutional operations is fused to form a new feature map and stitched, at which time the number of channels becomes twice as many as the original one, fusing feature information of different complexity. Then, it is fused with the output feature map after 3 × 3 convolution, batch normalization, and ReLU operation, and finally, the feature map is input to the next residual model. The residual module introduced in this module enhances the feature extraction capability at different depths; two 5 × 5 depth convolutional layers are able to extract semantic information at different levels of complexity, which are adopted to the feature extraction stage of the high-level feature map. So, this module is used instead of the convolutional layer in the original Attention U-Net network.

#### 2.2.3. Hybrid Loss Function

The loss function optimizes the network structure by backpropagating the numerical error of the calculated loss function and continuously updating the weights. In the field of medical image segmentation, the Dice Loss [45] function is commonly used to calculate the degree of differences between the predicted region and the real region. The concepts of Dice Loss are defined by (3). However, the loss function has the problem that the training error curve is very confusing when using the Dice Loss or IOU [46] loss function causes. These situations can be avoided by using the cross-entropy loss (CEL) function, with the expression (4), which makes the gradient form better, but suffers from the problem of class imbalance.

(3) $$\begin{array}{c} D=1-2\left|X\cap Y\right|/\left(\left|X\right|+\left|Y\right|\right)\text{,} \end{array}$$

(4) $$\begin{array}{c} CL=-\overset{n}{\underset{i=1}{\sum }}y_{i}^{c_{i}}\log\left(y_{i}^{{\hat{c}}_{i}}\right), \end{array}$$

where *X*∩*Y* denotes the intersection of *X* and Y; |X| and |Y| denote the number of *X* and Y, respectively; and *y*_*i*_^*c*_*i*_^and $y_{i}^{{\hat{c}}_{i}}$denote the label value and the predicted value, respectively.

To address the above problems, we use a hybrid loss function based on cross-entropy loss function and Dice loss function, which observes the convergence steadily during the training process and avoids the category of the imbalanced problem. Its formula is as follows:

(5) $$\begin{array}{c} DCL\left(Q,F\right)=-\frac{1}{U}\overset{H}{\underset{H=1}{\sum }}\overset{U}{\underset{U=1}{\sum }}\left(y_{n,l}\log p_{nl}+\frac{2y_{n,l}p_{nl}}{y_{n,l}^{2}p_{n,l}^{2}}\right), \end{array}$$

where *Q* is the actual situation, F is the predicted result from training, *y*_*n*,*l*_ ∈ *F* represents the probability of prediction, the *p*_*nl*_ ∈ *Q* represents the established target, H represents the number of classifications in the dataset, and U represents the number of pixels in the image.

## 3. Experiment

### 3.1. Experimental Data and Environment

The experimental dataset used in this study is collected from Qingdao Hospital of Shandong University Qilu Hospital. The collected data contain 420 MRI T1 images of 180 lumbar spine patients, which were expanded to 1000 images by data enhancement. In order to facilitate the later experimental operation, the dcm format of T1 images is changed to jpg format by the SimpleITK toolkit, and the image size is resized to 512 × 512 by linear interpolation. Finally, the images are classified into training sets, validation sets, and test sets according to the ratio of 7 : 2:1.

Experimental environment: Windows 10 operating system, AMD Ryzen 7 4800H processor, NVIDIA GeForce GTX 1650 GPU with 64 GB of video memory, 8-core CPU, 32 G of memory, PyTorch 1.2.0 is used for the deep learning framework, and Python is used for the programming language.

### 3.2. Data Preprocessing

The MRI images of the lumbar spine contain tissues such as vertebrae, intervertebral discs, spinal canal, and muscles.

**Figure 6:**
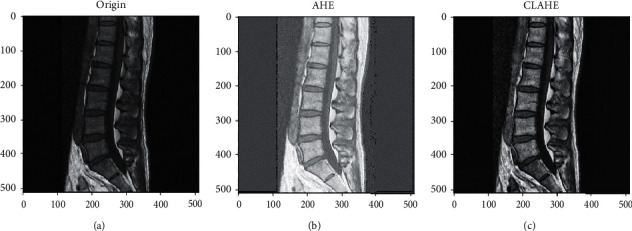
The comparison results of equalization. (a) Original graph. (b) Histogram equalization. (c) Our method.

The texture features of tissues reflect different basic feature information. Because the intervertebral disc in the original image is relatively dark and the contrast with the vertebral block is not obvious, it is easy to cause errors in the manual labeling of the intervertebral disc and vertebral block in the later stages, making the labeled image inaccurate and affecting the effect of later deep learning. Therefore, in order to facilitate labeling, the contrast of different tissues in the image needs to be improved. The histogram equalization method can increase the gray value range of the image and uniformize the distribution of pixel gray values, thereby improving the contrast and clarity of the image. The specific steps are as follows: first, the grayscale values are calculated, and the histograms are counted. Then, the cumulative histogram in the statistical histogram is calculated, and finally, the interval conversion is performed on the cumulative histogram. The effect is shown in Figure 6(b). The figure shows that after the histogram transformation of the whole image by the conventional histogram equalization operation, the brightness of the image is improved, the image noise is amplified, and even some parts appear. The effect of excessive brightness is that it eventually leads to a decrease in the sharpness of the image.

In order to effectively solve the problem of the image noise signal being amplified, a method of limiting contrast is added to the adaptive histogram equalization: if the value in a certain range of the histogram exceeds the limited threshold, the exceeding area will be cut out and that area is distributed to the rest of the histogram. As shown in Figure 6, compared with conventional histogram equalization, the method used in this study has a better processing effect and improves the contrast and sharpness of different tissues.

**Figure 7:**
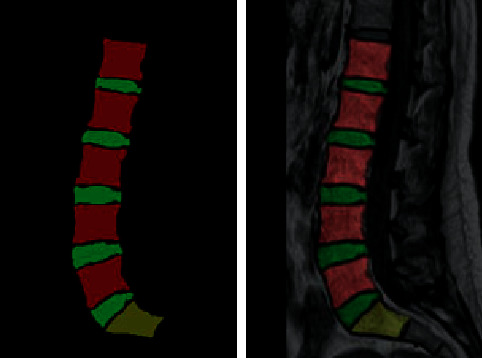
Marked effect.

The processed images are labeled and classified by the LabelMe tool; each image is labeled with 3 categories, including 5 vertebral bodies, 5 intervertebral discs, and 1 sacrum, named L, LD, and S. The different tissues labeled are distinguished by different colors, as shown in Figure 7. After the image is annotated, the corresponding JSON format file is obtained, which contains information about the different categories of annotation and the pixel coordinates of the annotation points. The JSON file is transformed into voc data, which contain the segmented mask map, the annotation map combining the mask map with the original map, and the NPY format file.

### 3.3. Evaluation Indicators

To comprehensively evaluate the effectiveness of the proposed algorithm for lumbar spine segmentation, three metrics are used as evaluation methods, which are accuracy (P), recall (R), and Dice similarity coefficient. As shown in formulas (6) and (7), when the accuracy, P, is high, the recall, *R*, is low and vice versa. When the two do not conform to this relationship, the Dice similarity coefficient is introduced for comprehensive evaluation. The Dice similarity coefficient is often used to evaluate medical image targets with uneven segmentation size. The obtained formula is (8).

(6) $$\begin{array}{c} P=\frac{TP}{\left(TP+FP\right)}\text{,} \end{array}$$

(7) $$\begin{array}{c} R=\frac{TP}{\left(TP+FN\right)}\text{,} \end{array}$$

(8) $$\begin{array}{c} Dice=\frac{2TP}{\left(TP+FP+TN+FN\right)}\text{,} \end{array}$$

where true positive (TP) is the number of lumbar spine images that the model correctly classifies as positive examples, that is, the number of samples that are actually positive examples and are classified as positive examples by the model. False positives (FP), which indicates the number of lumbar spine images that the model incorrectly classifies as positive, that is, the number of samples that are actually negative but are classified as positive by the model. True negative (TN) is the number of lumbar spine images correctly classified as negative by the model, that is, the number of samples that are actually negative and are classified as negative by the model. False negative (FN), which indicates the number of lumbar spine images that the model incorrectly classifies as negative examples, that is, the number of samples that are actually positive examples but are classified as negative examples by the model.

## 4. Results and Analysis

### 4.1. Attention U-Net Segmentation Effect

**Figure 8:**
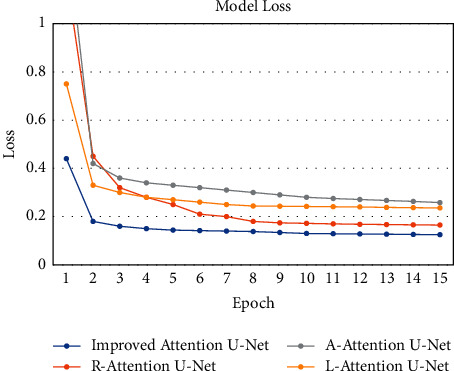
The loss curve.

During the training of the algorithm model, the batch size is set to 2, and 15 rounds are trained with one validation per round as well as model preservation. In this paper, the models of the Attention U-Net network with improved residual module, attention mechanism, and hybrid loss function are defined as R-Attention U-Net, A-Attention U-Net, and L-Attention U-Net, respectively. As shown in Figure 8, with the continuous iteration of R-Attention U-Net, A-Attention U-Net, L-Attention U-Net, and the improved model in this study, the loss function gradually decreases. At the beginning of training, after three rounds, the loss function of all models rapidly decreases to below 0.4. After the end of training, the loss functions of all models were stabilized below 0.3, and segmentation models satisfying the requirements were obtained.  Table 1 shows the experimental results of different models for lumbar spine detection. The accuracy rates of R-Attention U-Net, A-Attention U-Net, and L-Attention U-Net models are all above 90%, which indicate that the methods for improving a single variable all have better performance. Among them, A-Attention U-Net has the highest recall rate of 95.70%, but the number of incorrectly identified samples is high, resulting in a low accuracy rate. The improved Attention U-Net in this study has a lower recall rate compared with the A-Attention U-Net with the improved attention mechanism only, but the accuracy and Dice similarity coefficient are improved by 2.23% and 0.54%, respectively, i.e., the incorporated residual module and hybrid loss function have a better correction effect for the incorrect identification of the A-Attention U-Net.

**Figure 9:**
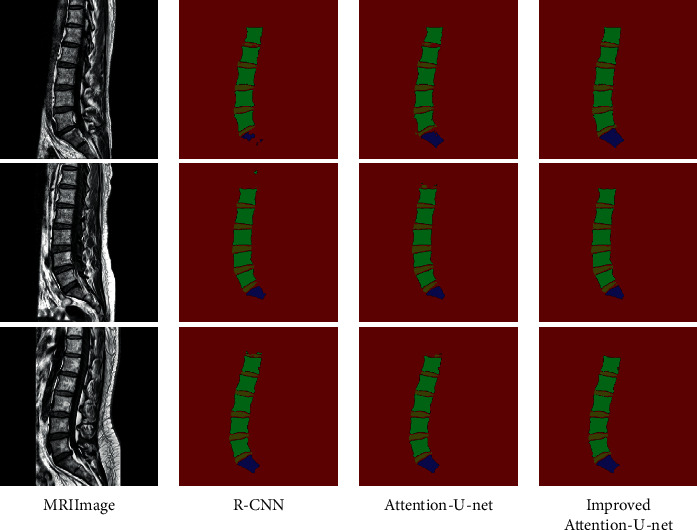
The segmentation effect of different algorithms.

Table 2 shows the comparison of experimental results under equal conditions; SVM, FCN, R–CNN, U-Net, Attention U-Net, and improved Attention U-Net are used for samples segmentation, respectively; the improved model in this study achieves the best results in terms of accuracy, recall, and Dice similarity coefficient indexes, which are 95.50%, 94.53%, and 95.01%, proving that the proposed method meets the requirements of lumbar spine segmentation. Among them, R-CNN and Attention U-Net also have better recognition effects, and by comparing the experimental results of these two models and the method in this study, as shown in Figure 9, the segmentation effect of the algorithm in this study is significantly better than other methods in terms of overall and detail processing. The sacrum and intervertebral disc appear missing and broken by R-CNN and Attention U-Net processing, while the proposed algorithm can segment the feature edges of the target more accurately, reducing the occurrence of problems, such as the mutilation of the intervertebral disc and the missing sacrum, and showing better robustness to targets with poor clarity and different shapes.

### 4.2. Postprocessing of Segmented Images

**Figure 10:**
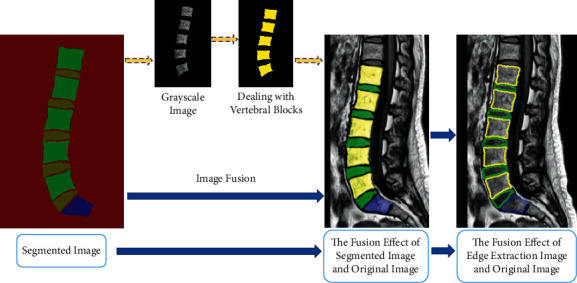
The segmentation effect of different algorithms.

Postprocessing of the images is required to observe the different parts of the lumbar spine more clearly. As shown in Figure 10, different categories are represented by different colors, with the vertebral block represented by green, the intervertebral disc represented by yellow, the sacrum represented by blue, and the background represented by red. The extraction of each category is performed according to the different colors of the segmentation object. First, the mask image is converted to a grayscale image, and then, the grayscale value threshold is set by the different grayscale value sizes of the vertebral block and intervertebral disc, so that the vertebral block and intervertebral disc can be extracted. Figure 10 shows the effect of extracting the vertebral block alone.

In order to realize the detailed view of different parts, we achieve the superposition of the original lumbar spine image and the mask image by image fusion and add labels to the hybrid map. Figure 10 shows the fusion map of the segmented images and the original image and the fusion map of the edge extraction and the original image, respectively. We can clearly see the various parts of the lumbar spine and lumbar disc from the fusion map, which is beneficial for the doctor to quickly diagnose the lumbar spine MRI images.

## 5. Conclusions and Future Work

Lumbar spine segmentation is very important for the diagnosis of related diseases. To address the problem of low segmentation accuracy of lumbar spine MRI images, we propose a segmentation method based on improved Attention U-Net. The steps of the study are as follows:

- Limiting contrast is added to the adaptive histogram equalization, which reduces the roughness of the image and improves the contrast and sharpness of different tissues, thus facilitating the labeling of experimental data
- By improving Attention U-Net, two residual modules are introduced instead of the original convolutional blocks, an attention module based on a multilevel feature map fusion is used, and a hybrid loss function is used in training for prediction
- Different tissues are extracted according to the different colors of the segmented images. And through image fusion, the superposition of the original lumbar spine image and the segmented image is realized, thus facilitating the physician to observe the lesion of each tissue more intuitively.

According to the comparison experiments of the three models with changing single variable, among them, the recall rate of A-Attention U-Net performs better than the improved method in this paper, reaching 95.70%, but the false recognition rate of A-Attention U-Net is higher, which leads to a decrease in accuracy and Dice similarity coefficient by 2.23% and 0.54%, respectively, proving that the method in this study is better than the improved single-variable method with better equalization ability. In addition, comparison experiments of six different network models were completed, and it was verified that the model in this study has better results in lumbar spine segmentation and outperforms SVM, FCN, R-CNN, U-Net, and Attention U-Net in terms of accuracy, recall, and Dice similarity coefficient, with 95.50%, 94.53%, and 95.01%, respectively. It proves that the method in this study has better performance in the intervertebral disc and more detailed processing of the sacral region with better robustness.

## Figures and Tables

**Table 1:** Comparison of experimental results of different improvement schemes.

Type	TP	FP	FN	P (%)	R (%)	Dice (%)
R-Attention U-Net	728	46	47	94.06	93.94	93.99
A-Attention U-Net	735	53	33	93.27	95.70	94.47
L-Attention U-Net	722	49	50	93.64	93.52	93.58
Improved Attention U-Net	743	35	43	95.50	94.53	95.01

**Table 2:** Comparison of experimental results of different algorithms.

Type	TP	FP	FN	P (%)	R (%)	Dice (%)
SVM	605	101	115	85.69	84.03	84.85
FCN	644	83	94	88.58	87.27	87.91
R-CNN	712	58	51	92.50	93.32	92.89
U-Net	685	62	74	91.70	90.25	90.10
Attention U-Net	715	49	57	93.59	92.62	93.10
Improved Attention U-Net	743	35	43	95.50	94.53	95.01

## Data Availability

The datasets used to support the findings of this study are available from the corresponding author upon request.
